# Intrinsic Reactivity of the Elusive Cyclic *N*,*N*‑Diacyliminium Ions in Mannich Reactions: The Complete Reactivity Order for Non, *N*‑Mono and *N*,*N*‑Diacyliminium Ions

**DOI:** 10.1021/acs.joc.6c00237

**Published:** 2026-08-04

**Authors:** Luiz Alberto B. de Moraes, Adriano Reis, Marcelo G. Montes D’Oca, Cristina M. Schuch, Ronaldo A. Pilli, Rodinei Augusti, Marcos N. Eberlin

**Affiliations:** † Mass Spectrometry Laboratory, Department of Chemistry, 28133 University of Sao Paulo, Ribeirao Preto, Sao Paulo 14040-901, Brazil; ‡ PPGEMN, School of Engineering, 42524 Mackenzie Presbyterian University and MackGraphe - Mackenzie Institute for Research in Graphene and Nanotechnologies, Mackenzie Presbyterian Institute, São Paulo, São Paulo 01302-907, Brazil; § Kolbe Laboratory of Organic Synthesis, Department of Chemistry, Federal University of Paraná, Curitiba, Paraná 81531-990, Brazil; ∥ Institute of Chemistry, 28132 University of Campinas, Campinas, São Paulo 13083-970, Brazil; ⊥ 28114 Federal University of Minas Gerais, Department of Chemistry, Belo Horizonte, Minas Gerais 31270-901, Brazil

## Abstract

We report the first intrinsic reactivity measurements of four cyclic *N,N*-diacyliminium ions in Mannich-type reactions. Using MS^n^ experiments (n = 1, 2) performed in the unique, convenient, and gaseous environment of a pentaquadrupole mass spectrometer, we reacted four gaseous five- or six-membered cyclic *N,N*-diacyliminium ions with allyltrimethylsilane and have established a complete reactivity order, demonstrating that *N,N*-diacyliminium ions surpass their *N*-monoacyl counterparts in reactivity. The study also enabled the isolation and characterization of key Mannich intermediates, confirming their bound nature and validating long-standing mechanistic proposals. Calculations have also indicated that the Mannich intermediates for the *N,N*-diacyliminium ions undergo prompt cyclization, and that therefore the reactions of these *N,N*-diacyliminium ions with an exocyclic *N*-acyl group with allyltrimethylsilane form polar [4^+^ + 2] cycloadducts. These findings provide a fundamental, solvent-free framework for understanding the electrophilic behavior of these key organic ions, completing the reactivity scale for cyclic nonacyl, *N*-mono-, and *N,N*-diacyliminium ions.

## Graphical abstract

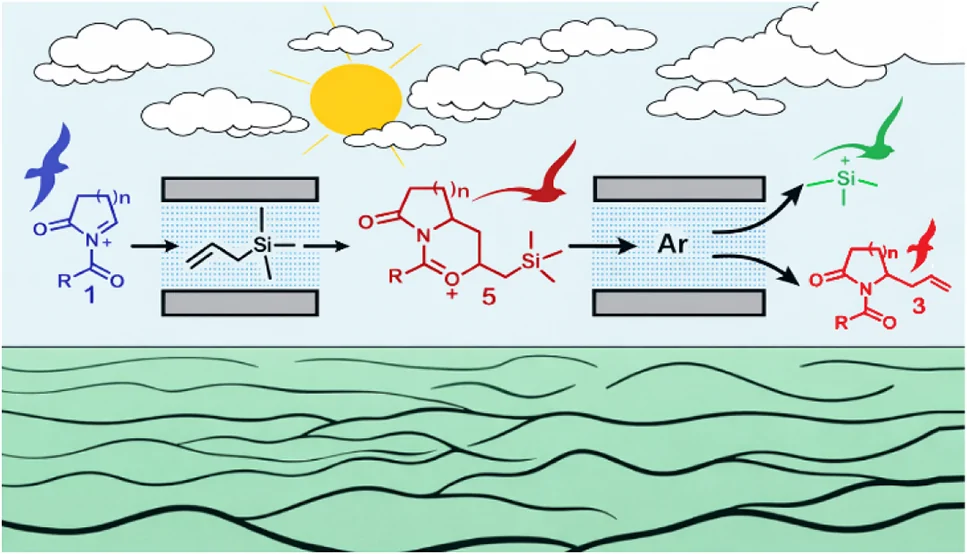

## Introduction

Two and a half decades ago, using a pentaquadrupole mass spectrometer,^1^ a unique and complete laboratory to study the intrinsic, solvent- and counterion-free reactivity of key ionic species in organic reactions (Figure 1),^2^ we have studied the intrinsic electrophilic reactivity of cyclic *N*-monoacyliminium ions in Mannich-type reactions.^3^ These ions are very versatile intermediates for the synthesis of a great variety of organic molecules, such as heterocycles and peptides, undergoing numerous types of reactions, such as nucleophilic additions, cyclizations, and rearrangements.^4^

**Figure 1:**
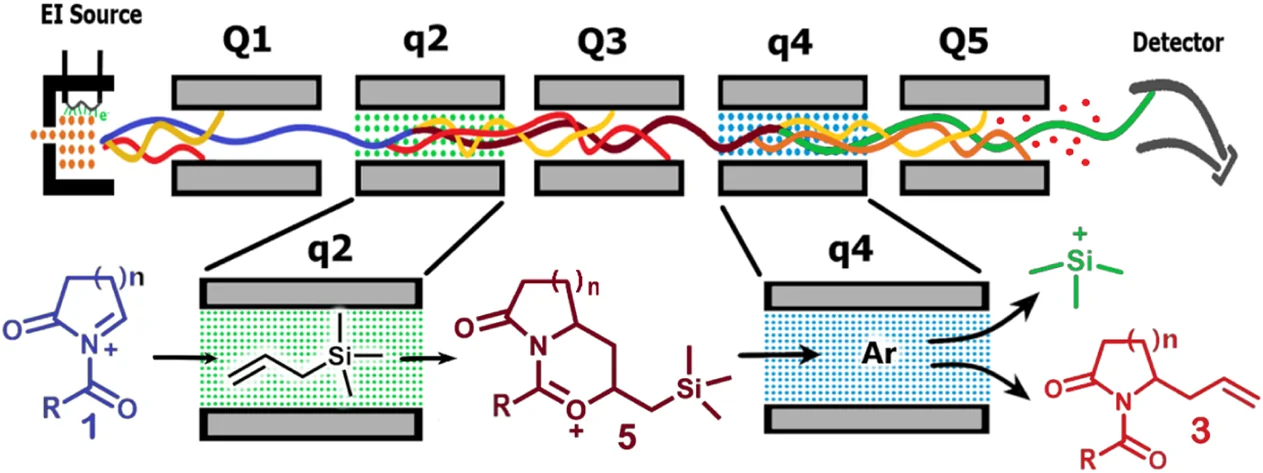
Schematics of the MS^n^ (*n* = 1, 3) experiments that can be performed in a pentaquadrupole mass spectrometer to study the intrinsic solvent- and counterion-free reactivity of key organic ions. The reactivity of the blue *N,N*-diacyliminium ions **1** (Scheme 1) with ATMS, forming purple intermediates **5** (Scheme 6eer) and then the final rgproducts **3** plus Si­(CH_3_)_3_^+^, is used to illustrate the steps and the processes happening inside each scanning (Q1, Q3, and Q5) and collision rf-only (q2 and q4) quadrupoles.

These versatile and readily accessible ionic intermediates have offered, therefore, powerful substrates for the controlled and efficient synthesis of diverse organic compounds.^2^ The tandem MS^2^ and MS^3^ experiments available in the unique pentaquadrupole gaseous environment have offered us a suitable laboratory to study the intrinsic (without the influence of solvents or counterions) reactivity of many fully isolated ions taking part as reactants or intermediates in key organic and organometallic reactions such as (trans)­acetalization,^5^ electrophilic aromatic nitration,^6^ polar [4 + 2^+^] cycloadditions,^7^ and novel and environmentally relevant ozone reactions.^8^

Using allyltrimethylsilane [CH_2_=CH–CH_2_–Si­(CH_3_)_3_, ATMS] as a model nucleophile and pentaquadrupole MS^*n*^ (*n* = 1, 3) experiments, we have established an intrinsic reactivity scale (Scheme 1) toward nucleophilic addition for five (**1a**–**f**) and six-membered (**1g**–**k**) cyclic nonacyl and *N*-monoacyliminium ions, either endo or exocyclic, and found this scale to match perfectly the solution behavior of the solvated ions.

**Scheme 1:**
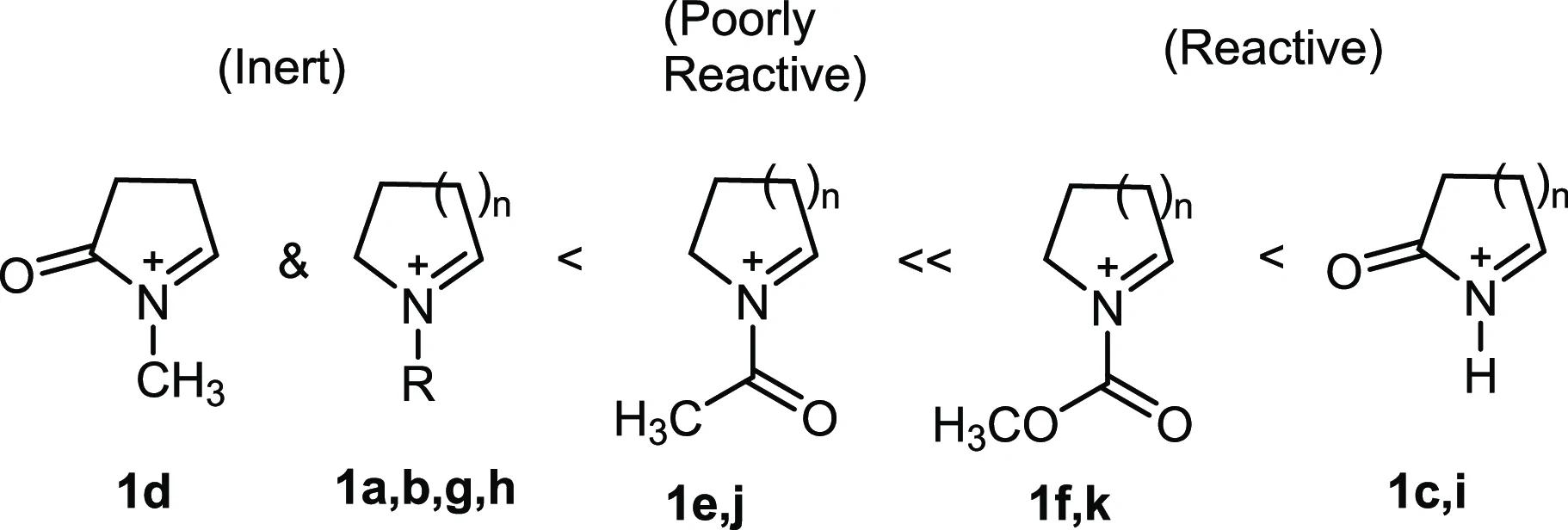
Intrinsic Reactivity Scale for Cyclic *Non*- and *N*-Monoacyliminium Ions

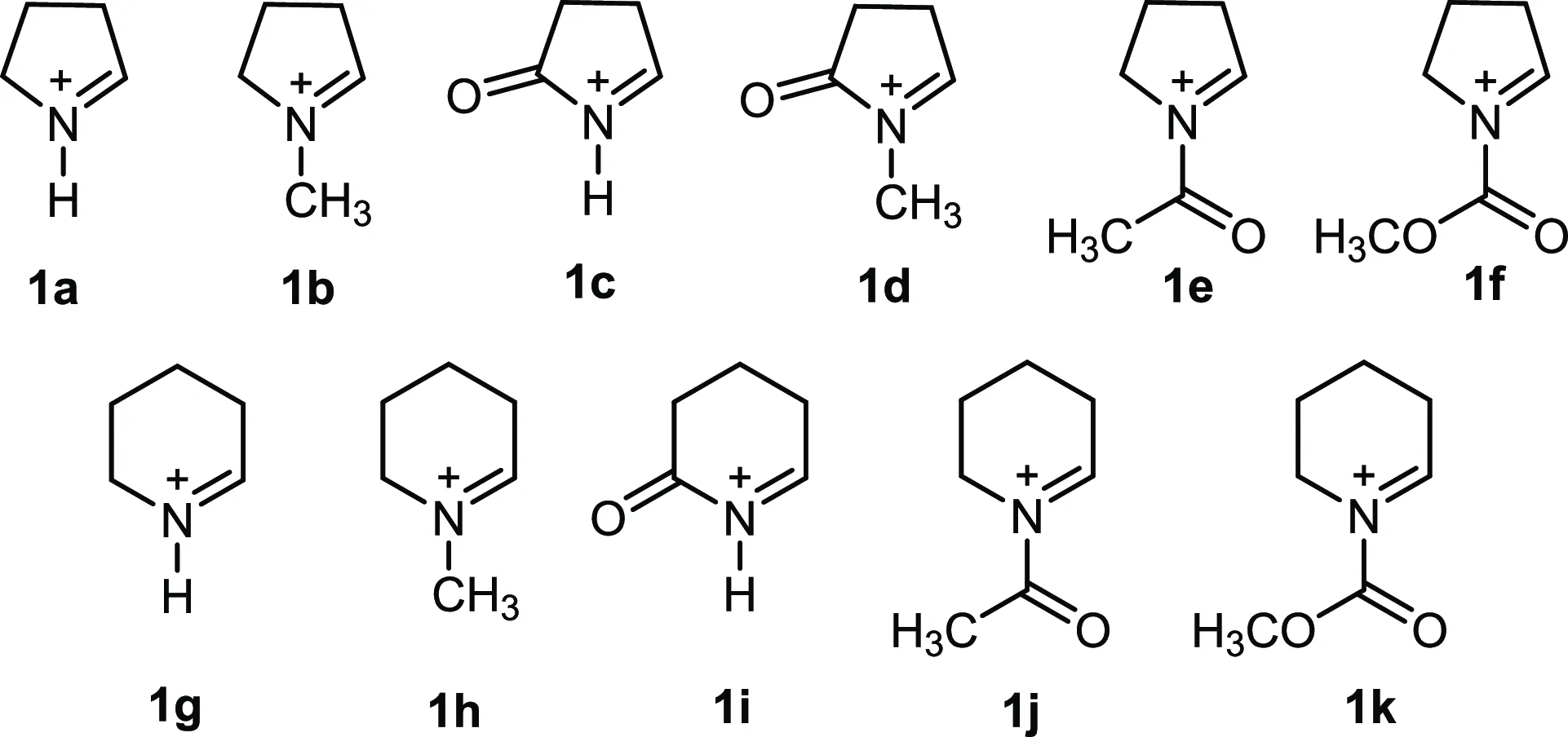



Our results showed, for instance, that *N*-alkyl (“non-acyl”) iminium ions lacking *N*-acyl activation (**1a,b**) are intrinsically inert toward nucleophile (ATMS) addition, confirming the critical role of *N*-acyl activation. Among the *N*-monoacyliminium ions tested, those bearing endocyclic, *s-trans*-locked *N*-carbonyl groups (**1c** and **1i**) were found to be the most reactive, with five-membered ring (n = 1) ions showing greater reactivity than their six-membered (n = 2) analogues. Notably for what could be taken as a nearly “innocent” group, *N*-methyl substitution in **1d** strongly suppresses reactivity, rendering **1d** fully inert despite its structural similarity (endocyclic *N*-carbonyl group) to the most reactive **1c**.

In summary, our findings and conclusions were as followed:^3^ we found that “non-acyl” *N*-alkyliminium ions (**1a,b,g,h**) are deactivated toward nucleophile addition since their C2 charges are diminished owing to effective delocalization with the adjacent nitrogen, whereas the reverse reaction (Grob fragmentation) is facilitated by the electron-rich nitrogen atom. The *N*-acyl groups of **1e,j**, **1f,k**, and **1c,i** disfavor Grob fragmentation and increase C2 electrophilicity via their resonance interaction with the nitrogen lone pair and electron-attracting inductive effects, thus favoring ATMS C2-addition. But facile rotation of the exocyclic N-acyl bond (rotation barriers of ca. 10 kcal/mol or less) minimizes these effects; hence, C2-activation is minor, and **1e,j** and **1f,k** are not very reactive toward ATMS addition.

The *N*-monoacyliminium ions **1c,i** with endocyclic N-acyl groups are, however, locked in planar *s-trans* conformations that favor N–C=O cross conjugation that reduces cation stabilization by the nitrogen electron pair; hence, ATMS addition at a more electrophilic C2 occurs readily.

Another novelty of our work was the first isolation (by quadrupole selection) and direct characterization (via MS^3^ experiments) of the key intermediates of gas-phase Mannich-type reactions, the gaseous β-silyl cation intermediates **2** (Scheme 2).

**Scheme 2:**
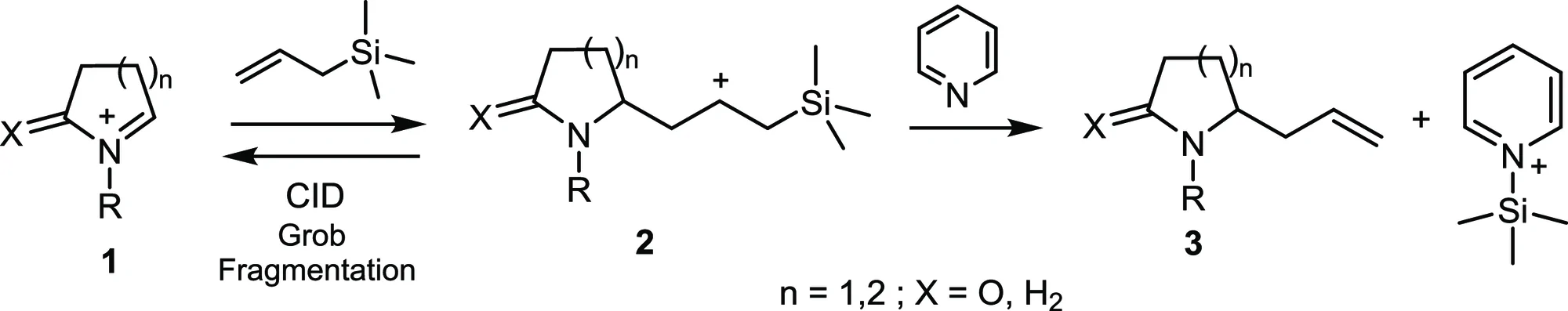
Sequential Reactions of *N*-Monoacyliminium Ions **1** with ATMS and Subsequent Reactions of Intermediates **2** with Pyridine That Forms the Mannich Intermediates **2** and the Final Mannich Product **3** ^a^

a Note that **2** seems to collapse to the polar [4^+^ + 2] cycloadduts **5**, see Scheme 7 and discussion that follows. Intermediates **2** dissociate exclusively upon collision-induced dissociation (CID) by Grob fragmentation.

To investigate the structures and reactivities of the key Mannich intermediates **2**, we used MS^3^ experiments to isolate and then either dissociate them via collisions with argon or react them with pyridine, which was used as a model nucleophile. Upon more energetic (15 eV) dissociative collisions with argon, **2** underwent Grob fragmentation reverting to the reactant ions **1** or, under lower energy collisions (near zero eV), reacted with pyridine via Si­(CH_3_)_3_^+^ abstraction (Scheme 2), validating the long-standing mechanistic proposals for Mannich reactions.

In a second MS^n^ pentaquadrupole study,^9^ we investigated the intrinsic gas-phase reactivity of three cyclic *N*-monoacyliminium ions **1c**, **1i,** and **1k** as well as a *N,N*-diacyliminium ion **1l** (Scheme 4) in Mannich-type reactions with vinyloxytrimethylsilane [CH_2_=CH–O–Si­(CH_3_)_3_]. We found that the two endocyclic *N*-monoacyliminium ions **1c** and **1i** react consecutively with two CH_2_=CH–O–Si­(CH_3_)_3_ molecules to form the stable adducts **4c** and **4i**, respectively (Scheme 3a). But the exocyclic *N*-monocarbomethoxy ion (**1k**) and a novel *N,N*-diacyliminium (**1n**) ion with both exo and endocyclic acyl groups, for which the *s-cis* conformation is favored, reacted by a novel polar [4^+^ + 2] cycloaddition yielding the stable, resonance-stabilized cycloadducts **5k** and **5n**, respectively (Scheme 3b). This report^9^ seems to be the initial one detailing a reaction involving an *N,N*-diacyliminium ion.

**Scheme 3:**
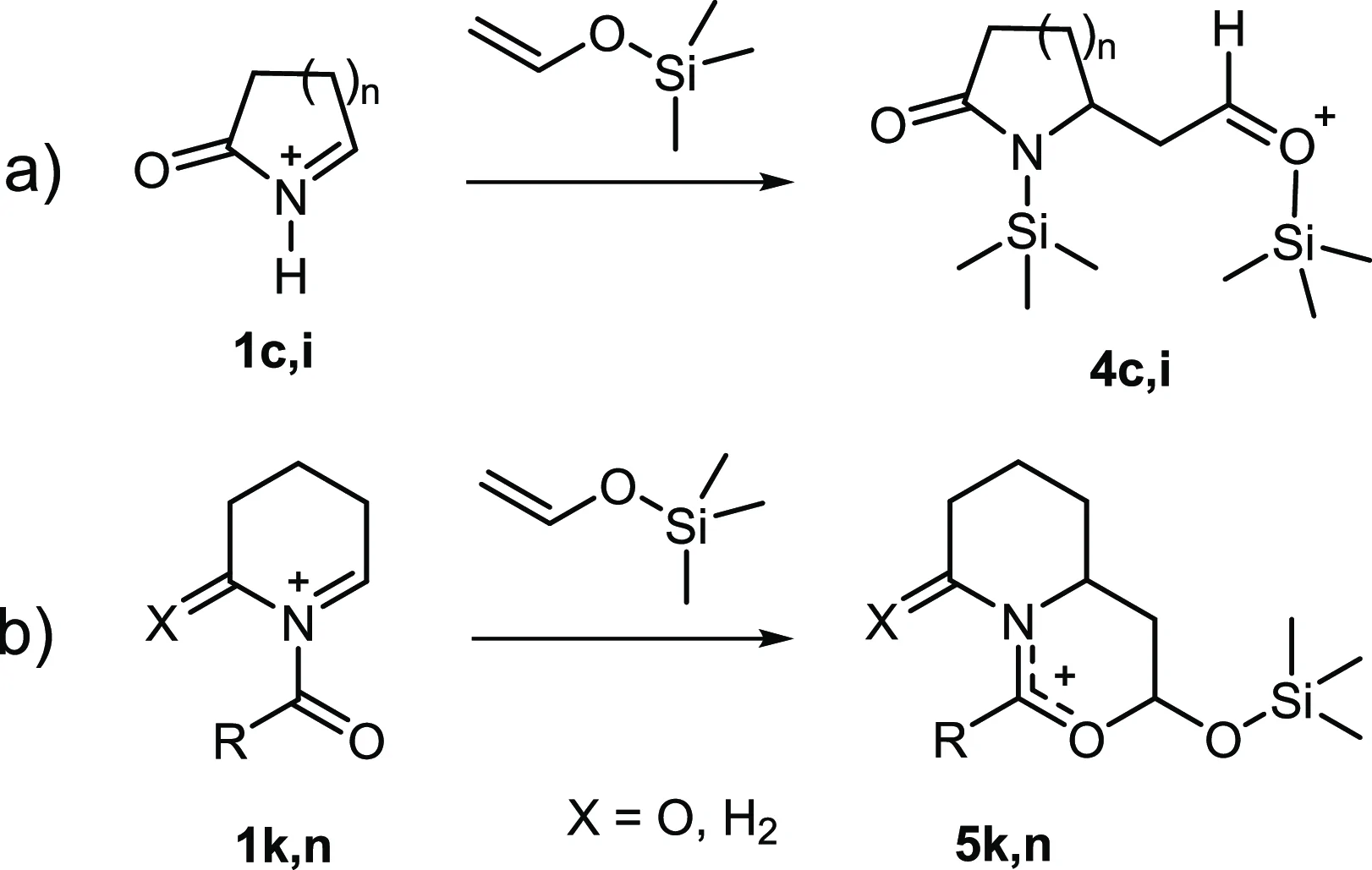
Reactions of a) the Endocyclic *N*-Monoacyliminium Ions **1c** and **1i** and b) the Exocyclic *N*-Monoacyliminium Ions **1k** (X = H_2_) and the *N,N*-Diacyliminium Ion **1n** with Vinyloxytrimethylsilane

Overall, these pioneering studies have demonstrated that the intrinsic reactivity of the gaseous *N*-monoacyl and *N,N*-diacyliminium ions closely mirrors solution trends, providing a fundamental and solvent-independent framework for monitoring the Mannich reaction flow and to understand more deeply *N*-acyliminium ion chemistry and mechanisms.^1^ But by the time of these first pioneering works, convenient precursors for cyclic *N,N*-diacyliminium ions, except for **1n**, were unavailable to us, hence these key ions were missing in our reactivity order (Scheme 1).

It would be important to investigate the reactivity of *N,N*-diacyliminium ions, since whereas the chemistry of *N*-monoacyl and *C,N*-diacyliminium ions are in general very well explored and extensively applied in synthesis and method developments, the subset of doubly acylated *N,N*-diacyliminium ions appear to be have been only incidentally cited as “broadly explored”,^10^ or only theoretically postulated^4^ or even cited as inaccessible^11^ owing to their high instability.^12^ Therefore, these potentially very reactive and synthetically attractive doubly acylated ions have remained essentially elusive both in solution and in the gas phase.

We have therefore concentrated our efforts on synthesizing suitable precursors for a representative set of *N,N*-diacyliminium ions and are glad to report that we succeeded. Using four of them (**6**, Scheme 4) and electron ionization, we have been able to form two five- (**1l,m**) and two six-membered (**1n,o**) cyclic endo/exo *N,N*-diacyliminium ions (Scheme 4), both in solution and as gaseous ions.

**Scheme 4:**
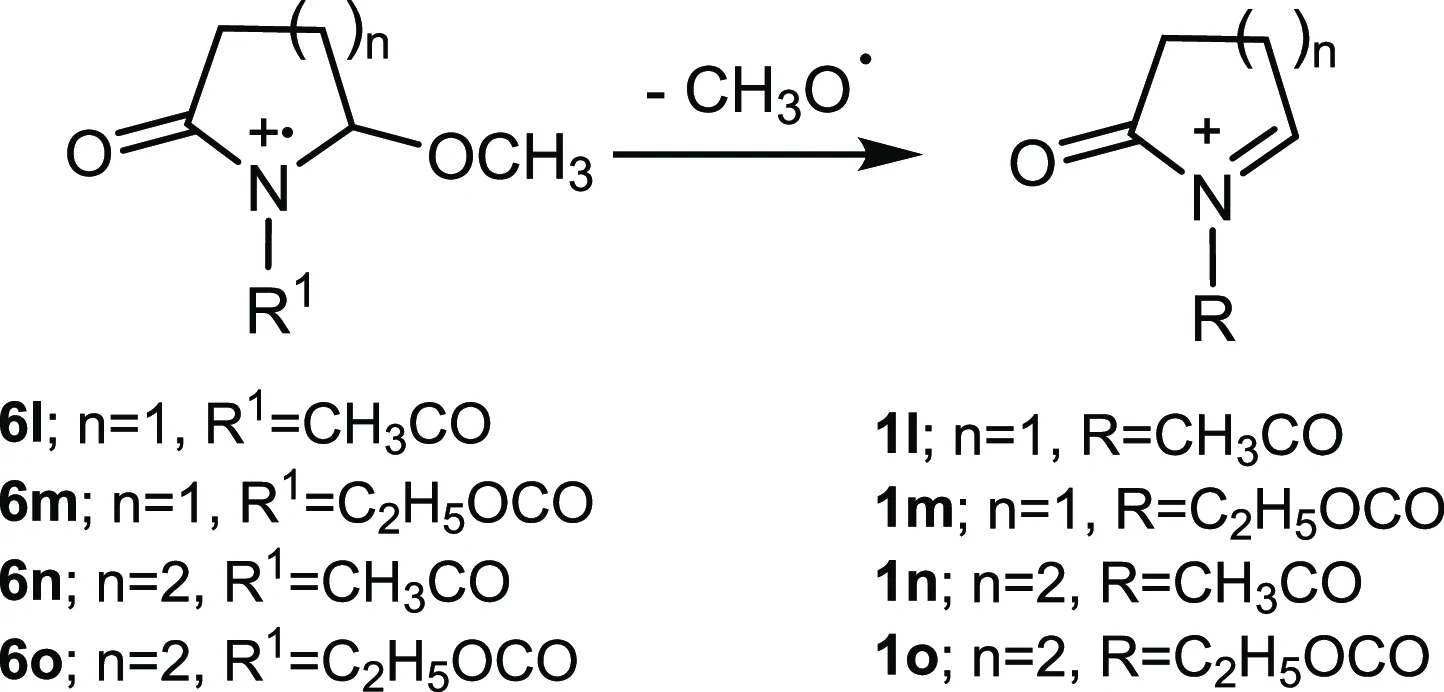
Formation of *N,N*-Diacyliminium Ions **1l**–**o** From 70 eV EI of Precursors **6l**–**o**

We report herein, therefore, the first measurements of the intrinsic reactivity of several cyclic *N,N*-diacyliminium ions (**1l**–**o**) in Mannich-type reactions, and present a complete order of intrinsic reactivity for cyclic iminium ions, now encompassing *non*-acyl, endo and exocyclic *N*-monoacyliminium ions (**1a,k**), and *N,N*-diacyliminium ions **1l**–**o**.

## Experimental Section

Double- (MS^2^) and triple-stage (MS^3^) mass spectrometric experiments,^14^ performed with an Extrel [Pittsburgh, PA] pentaquadrupole (Q1 q2 Q3 q4 Q5) mass spectrometer (Figure 1), were used to mass-select the gaseous ions **1l**–**o**, to react them with ATMS, and to characterize the structure of their product ions. Reactant ions **1l**–**o** were formed by 70 eV electron ionization (EI) followed by EI-induced hydrogen atom loss from the appropriate neutral precursors **6l,o** (Scheme 4), which were prepared by the NaBH_4_/MeOH–H^+^ reduction of the corresponding commercially available *N*-alkoxycarbonyllactams.^13^

Other sources describe how these precursors are prepared.^15^ The ion/molecule reactions were performed via MS^2^ experiments in which the ion of interest was mass-selected by Q1 and reacted further in q2 with neutral ATMS. Ion translational energies were set to near zero (ca. 1 eV) as calibrated by the *m*/*z* 39:41 ratio in neutral ethylene/ionized ethylene reactions.^14^ To record product ion mass spectra, Q5 was scanned while operating Q3 in the broad-band RF-only mode.

Multiple low-energy collisions that caused typical beam attenuations of 50–70% were used in q2 to increase reaction yields and to concomitantly promote collisional quenching of both the reactant and product ions. For the MS^3^ experiments, a q2 product ion of interest was mass-selected by Q3 and further dissociated by 15 eV collisions with argon, while scanning Q5 to acquire the product ion mass spectrum. The 15 eV collision energies were taken as the voltage difference between the ion source and the collision quadrupoles. The indicated pressures in each of the differentially pumped regions were typically 2 × 10^–6^ (ion source), 8 × 10^–6^ (q2), and 8 × 10^–5^ (q4) Torr. Further experimental details are described elsewhere.^1^ Semiempirical calculations were run on Spartan 14 (Wavefunction Inc.).

## Results and Discussion

Figure 2 illustrates, for ion **1m**, the sequential mass spectra recorded from the MS, MS^2^, and MS^3^ experiments used to evaluate the intrinsic reactivity of the *N,N*-diacyliminium ions **1** toward ATMS. These spectra also illustrate the suitability of the pentaquadrupole setup for serving as a complete and effective laboratory for studying such reactivities. Note that in this setup, quadrupoles Q1, Q3, and Q5 function as either ion transmission or selection devices, whereas q2 and q4 function as chemical reactors.

**Figure 2:**
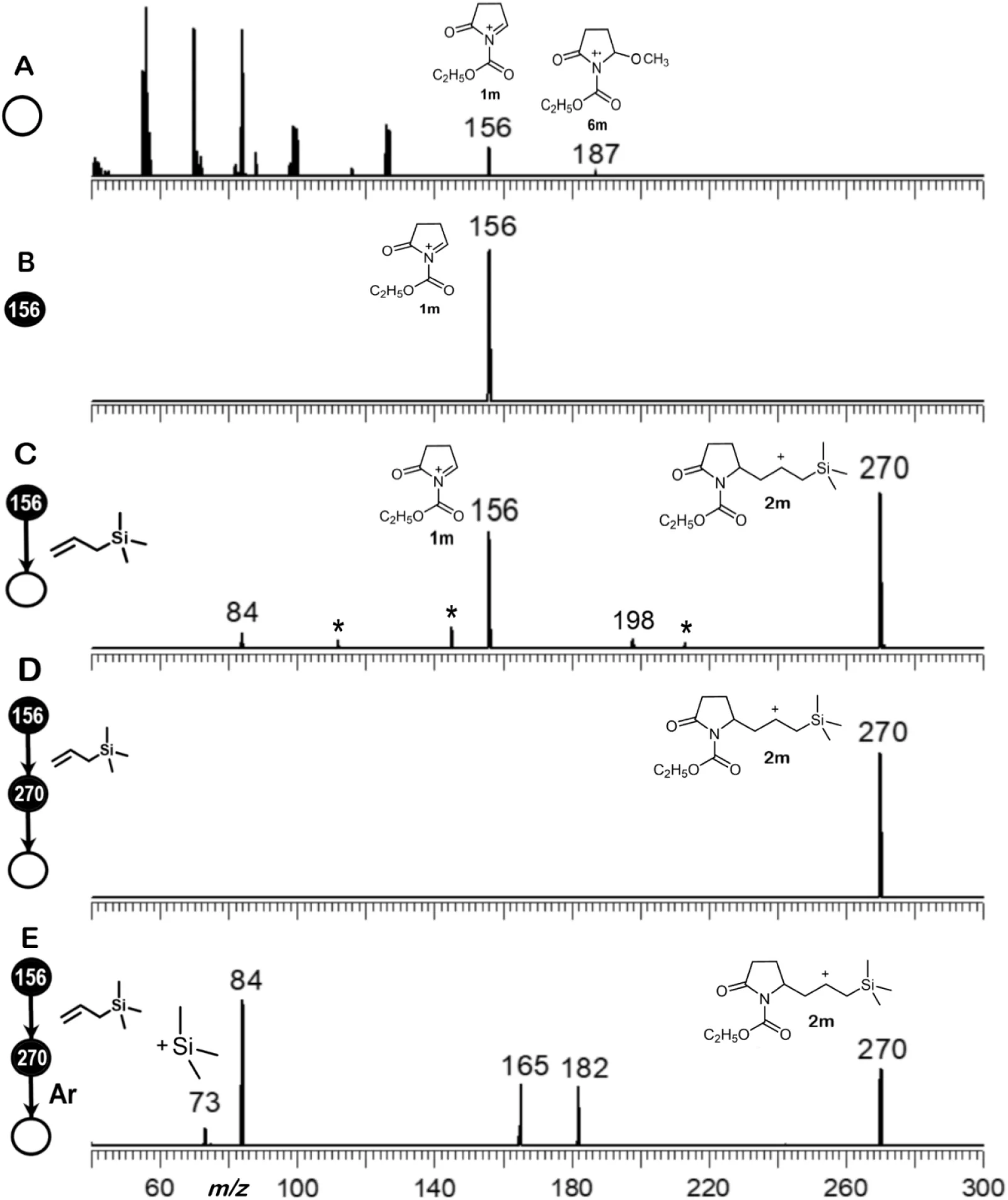
Illustrative sequential MS^n^ (*n* = 1, 3) collected during A-E experiments that are used to evaluate the intrinsic reactivity of the cyclic *N,N*-diacyliminium ion **1m** of *m*/*z* 156 toward ATMS.

A suitable gaseous precursor **6m** is introduced into the ionization source (Figure 2A), and 70 eV electron ionization then forms the molecular ion of **6m** of *m*/*z* 187, along with the desired *N*,*N*-diacyliminium ion **1m** of *m*/*z* 156 as a fragment. Q1 then mass-selects **1m** of *m*/*z* 156 (Figure 2B), and the isolated, gaseous, solvent and counterion free ion **1m** is subsequently transmitted to q2. Next, the isolated **1m** is exposed to ATMS, suffering low-energy collisions with it, and these near zero-eV collisions promote both cooling of **1m** and its reactions with ATMS to form the Mannich-intermediate **2m** of *m*/*z* 270 as a major product (Figure 2C).

In the following step (Figure 2D), Q3 mass-selects **2m** and transmits it to q4 where **2m** is structurally characterized via more energetic 15 eV collisions with argon. Finally, the fragment ions of **2m** are detected (Figure 2E). Note that since there should be no substantial discrimination of ion abundances for quadrupole ion transmission, signal intensities could be directly translated to product yields.

Surprisingly, if we consider the general behavior of the Mannich intermediates **2** from the reactions with the *N*-monoacyliminium ions, intermediate **2m** of *m*/*z* 270 suffers no Grob fragmentation^15^ at all back to **1m** of *m*/*z* 156 (Figure 2E), yielding instead several other fragment ions. The absence of reversibility of **2m** to reactants **1m** and ATMS emphasizes a covalently bound nature for **2m** while eliminating the loosely bound adduct.

A series of secondary ATMS products of *m*/*z* 73, 99, 145, 187, 213 and 239, likely formed via initial proton transfer, were also observed to compete with the Mannich reaction, but in solution, proton transfer is expected to be reversible (Scheme 2) and therefore to contribute less or to be irrelevant.

Similar MS^n^ experiments as those performed for **1m** were also performed for the other *N,N*-diacyliminium ions **1l**, **1n,** and **1o**. To better compare their relative reactivities toward ATMS, separate MS^2^ were collected for their ATMS reactions in q2 using the same collision conditions, such as collision energy and ATMS pressures, to ensure the best possible comparison.^6^ Then, the four MS^2^ data were combined in Figure 3 to show only the remaining reactant ion **1l**–**o** and their corresponding products **2l**–**o**. Note that the partial conversion to products is just a consequence of adding limited amounts of the ATMS reactant to the q2 reactor, to facilitate reactivity measurements, but that full conversion of **1l**–**o** to **2l**–**o** could be accomplished if desired by increasing the ATMS pressure.

**Figure 3:**
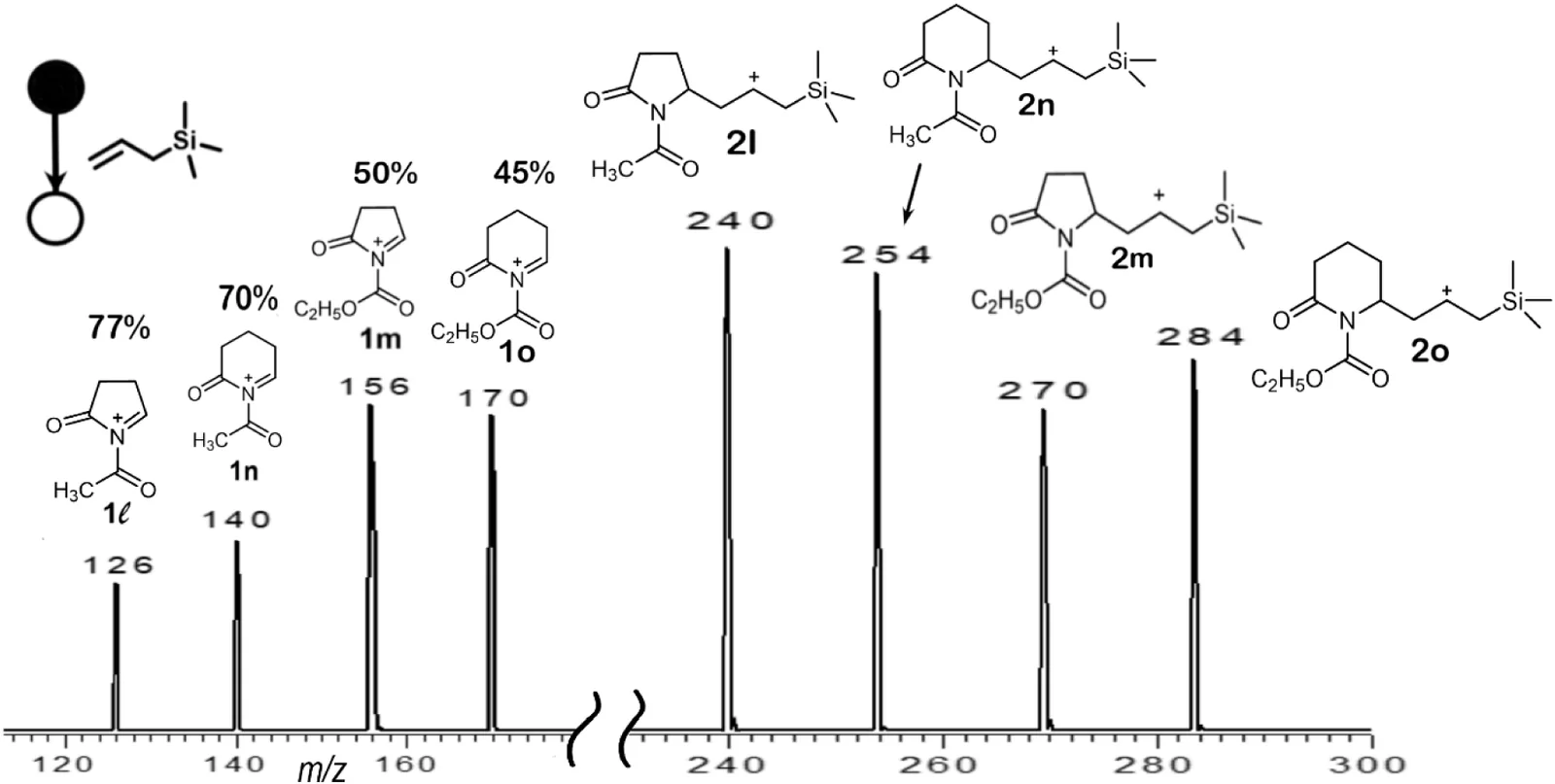
Combined partial MS^2^ for the reactions of the four *N,N*-diacyliminium ions **1l**–**o** with ATMS.

To compare reactivities, the virtual reaction yields measured by the abundances of product versus the initial reactant ion, that is [**2**]/[**2**] + [**1**] were also added to Figure 3. Both the five- (**1l**, 77%) and six-membered (**1n**, 70%) cyclic *N,N*-diacetyliminium ions were found to be considerably more reactive than their *N*-carboethoxy counterparts **1m** (50%) and **1o** (45%). As for five- versus six-membered rings, the five-membered *N*-COCH_3_ ion **1l** (77%) was more reactive than its six-membered analog **1n** (70%), whereas an inverted order of reactivity was observed for the *N*-CO_2_C_2_H_5_ ions **1m** (50%) and **1o** (45%). But these “five-versus-six” differences in reactivity, although perceptible, fall within (or are close to) experimental error.

A closer look at Figure 2C reveals that another key process happens in q2. Upon near zero eV collisions with ATMS, **1m** of *m*/*z* 156 also fragments losing CH_2_=CH_2_ and CO_2_ (Scheme 5) to form the mono *N*-acyliminium ion **1c** of *m*/*z* 84, which in turn reacts with ATMS, still inside q2, to form the Mannich intermediate **2c** of *m*/*z* 198. This unique scenario gave us the opportunity to compare, under nearly exact the same reaction conditions, the reactivity of five-membered cyclic *N*-monoacyl- versus *N,N*-diacyliminium ions, as Figure 4 illustrates for **1l** (Scheme 5).

**Scheme 5:**
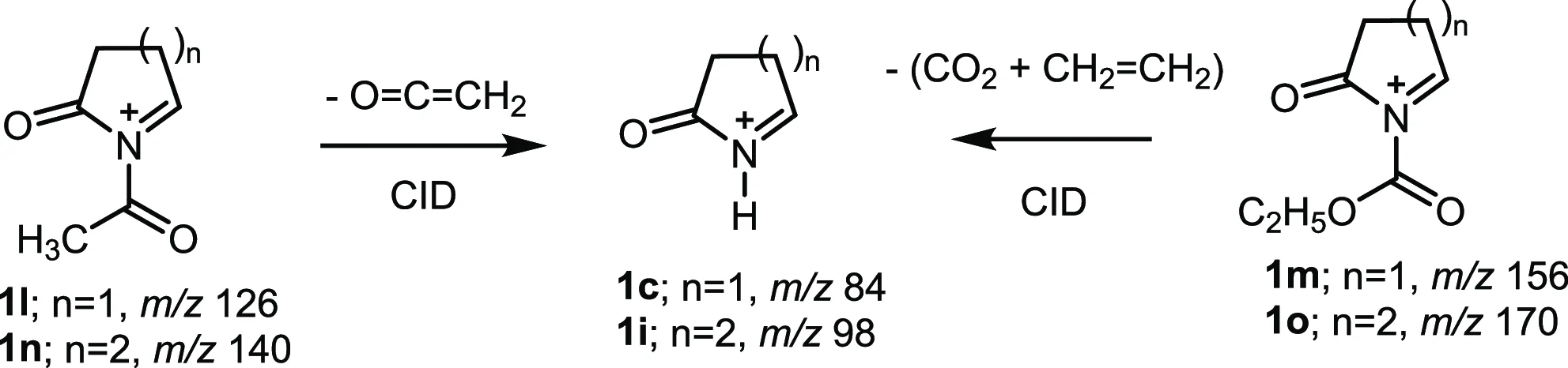
Collision-Induced Dissociation (CID) of the *N,N*-Diacyliminium Ions **1l**–**o** to *N*-Monoacyliminium Ions **1c,i** Occurring Inside q2

**Figure 4:**
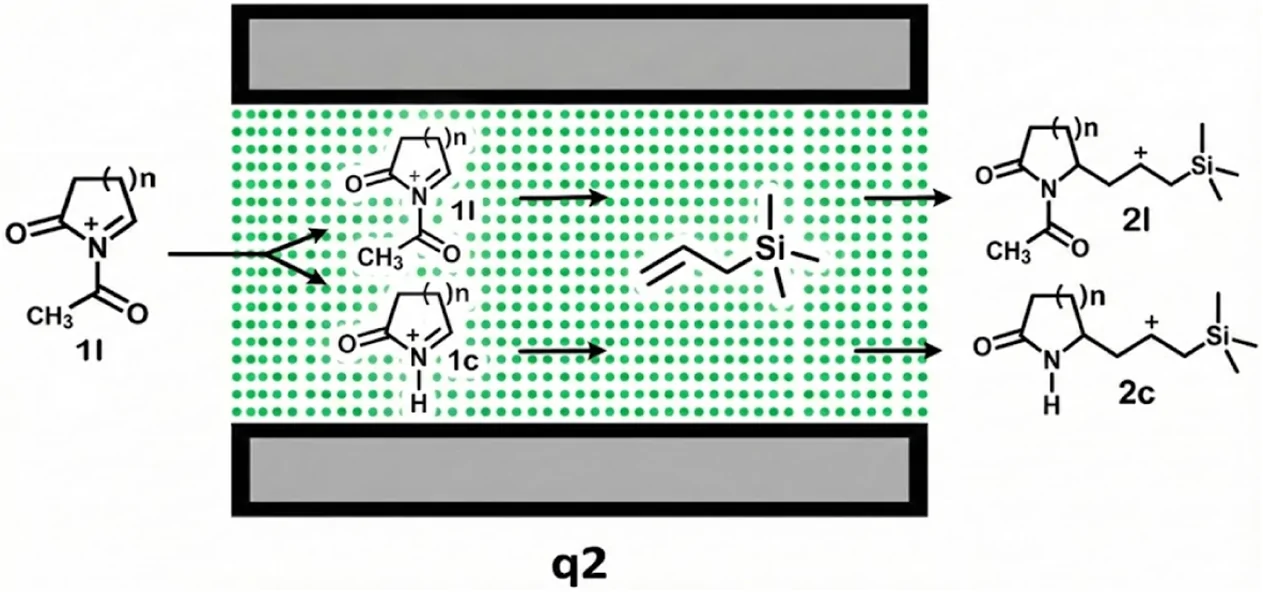
Schematic representation of dissociations/reactions occurring inside q2 using the *N,N*-diacyliminium ion **1l** as an example.

This trend was beneficial for reactivity investigations since it allowed us, via the very same tandem MS^2^ experiment, to form and immediately use, inside the same “chemical reactor” q1, pairs of *N,N*-diacyliminium ions with its homologue *N*-monoacyliminium ions, thus opening an unprecedented and best possible scenario to compare reactivities of *N*-mono versus *N,N*-diacyliminium ions (Figure 4).

Figure 5 shows the double-stage (MS^2^) product ion mass spectra collected for the reactions of the cyclic *N,N*-diacyliminium ions **1l**–**o** with ATMS under conditions (collision energy and pressure) finely adjusted to maximize both the formation of both the Mannich intermediates **2l**–**o** and the dissociation of the *N,N*-diacyliminium ions **1l**–**o** to their homologous *N*-monoacyliminium ions **1c** or **1i** (Scheme 5).

**Figure 5:**
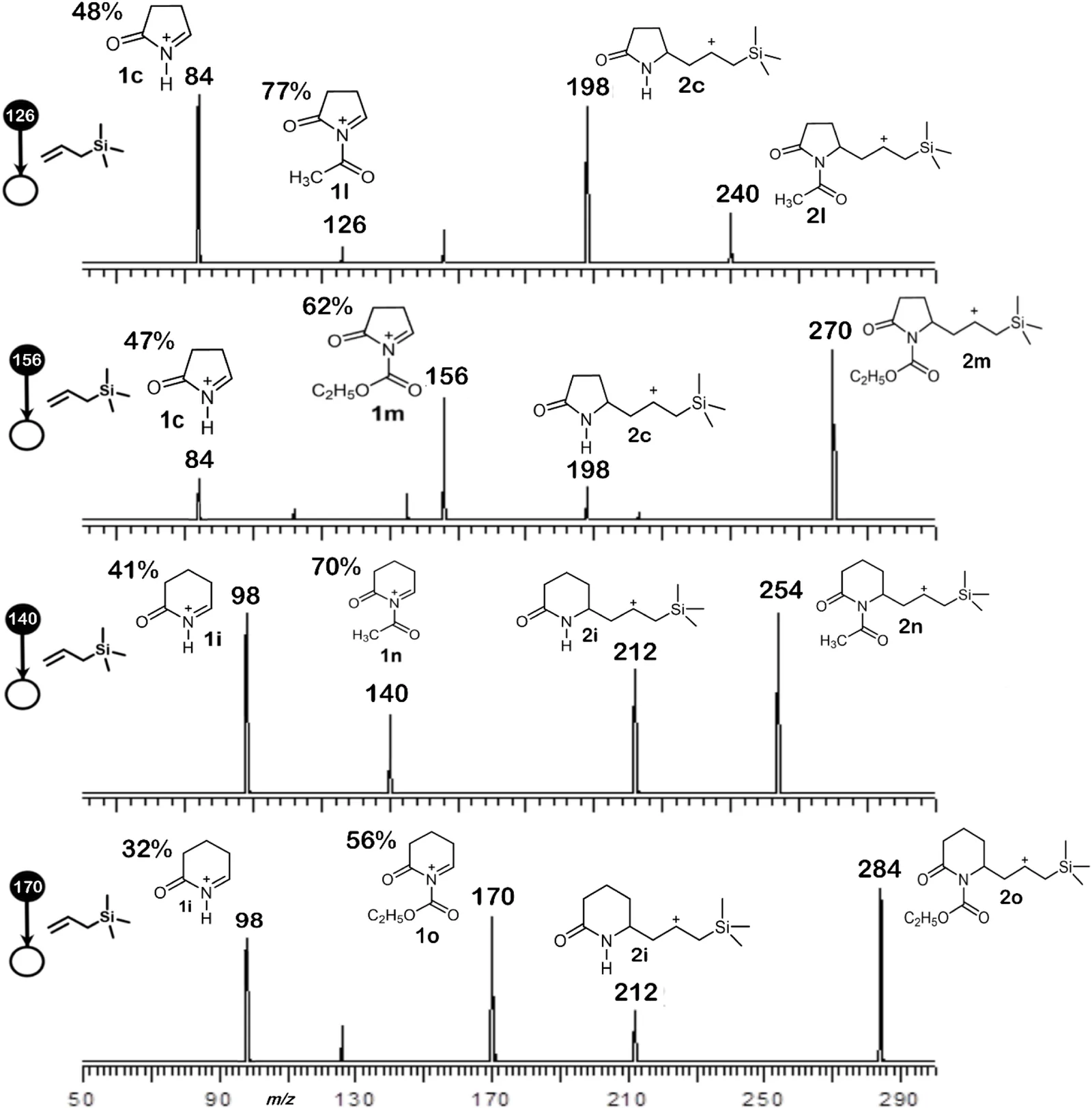
MS^2^ for the reaction of the *N,N*-diacyliminium ions **1l**–**o** with ATMS.

The yields of these reactions were also calculated and inserted into Figure 5 on top of each reactant ion **1l**–**o**. The presence of two activating acyl groups directly attached to N1 in the *N,N*-diacyliminium ions **1l**–**o** indeed results in superior C2-reactivity toward the ATMS nucleophile when compared to that of their *N*-monoacyliminium ions **1c** or **1i**. This greater reactivity is seen by comparing reaction yields **1l** (77%) versus **1c** (48%), **1m** (62%) versus **1c** (47%), **1n** (70%) versus **1i** (41%), and **1o** (56%) versus **1i** (32%).

Figure 6 compares, using **2c** and **2l** as examples, the MS^3^ for dissociation of the Mannich intermediates intercepted for the reactions of either the *N*-monoacyliminium ion **1c** or the *N,N*-diacyliminium ion **1l**. Note that **2c** predominantly undergoes Grob fragmentation to reform **1c** of *m*/*z* 84 (Scheme 6). Although anticipated for such class of ions, this classic fragmentation would still leave doubts on the covalently bound nature of **2c**, since such reverse-to-products fragmentation could be interpreted as an indication of a loosely bound ion/molecule complex. Figure S1 shows the MS^3^ of **2c** and **2l**, whereas Schemes S1–3 present our proposal for the dissociation of **2n**–**o**, in fact likely the [4^+^ + 2] cycloadducts **5n**–**o**.

**Figure 6:**
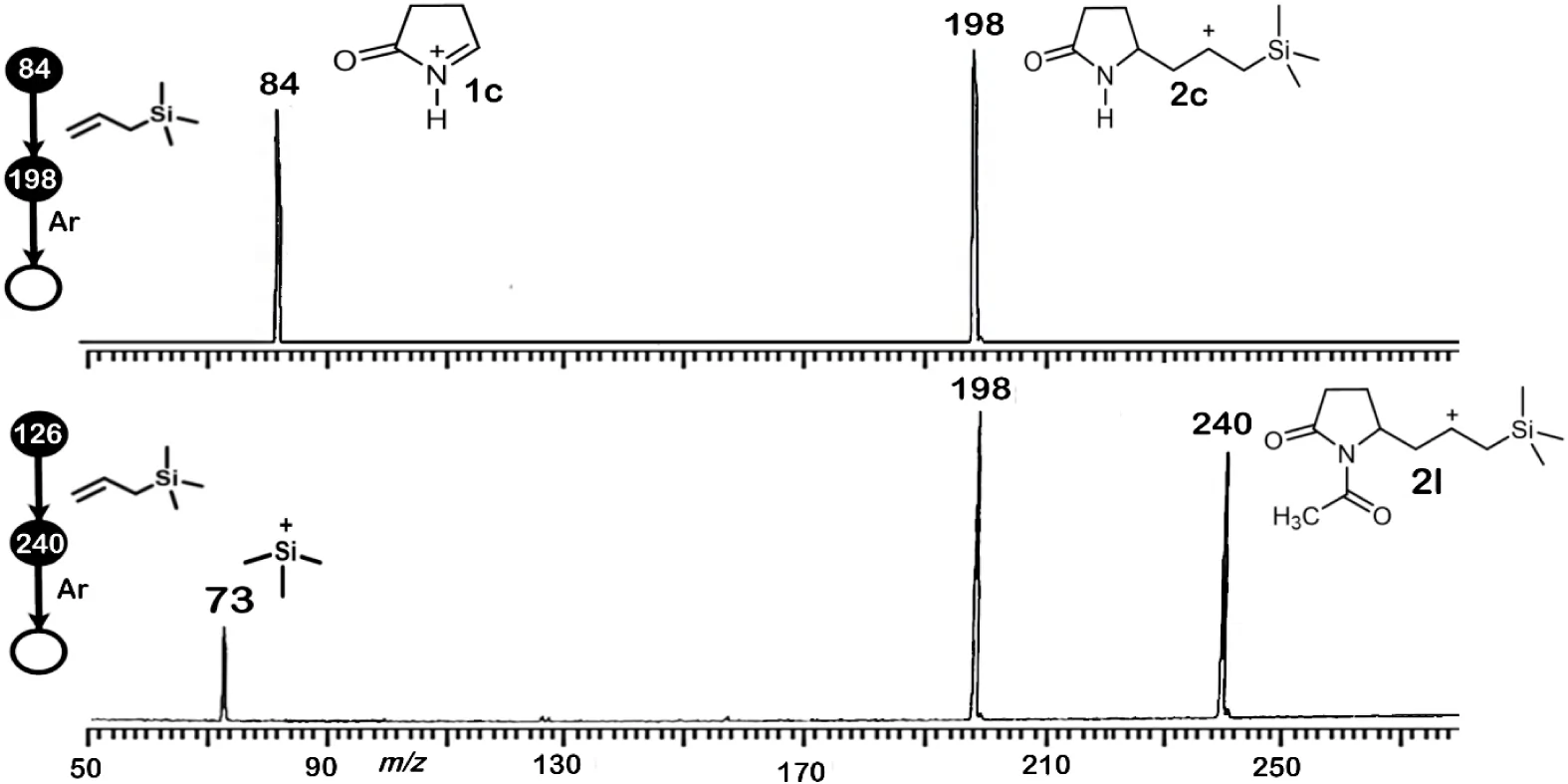
MS^3^ for dissociation of the Mannich intermediates **2c** of *m*/*z* 198 and **2l** of *m*/*z* 240.

**Scheme 6:**
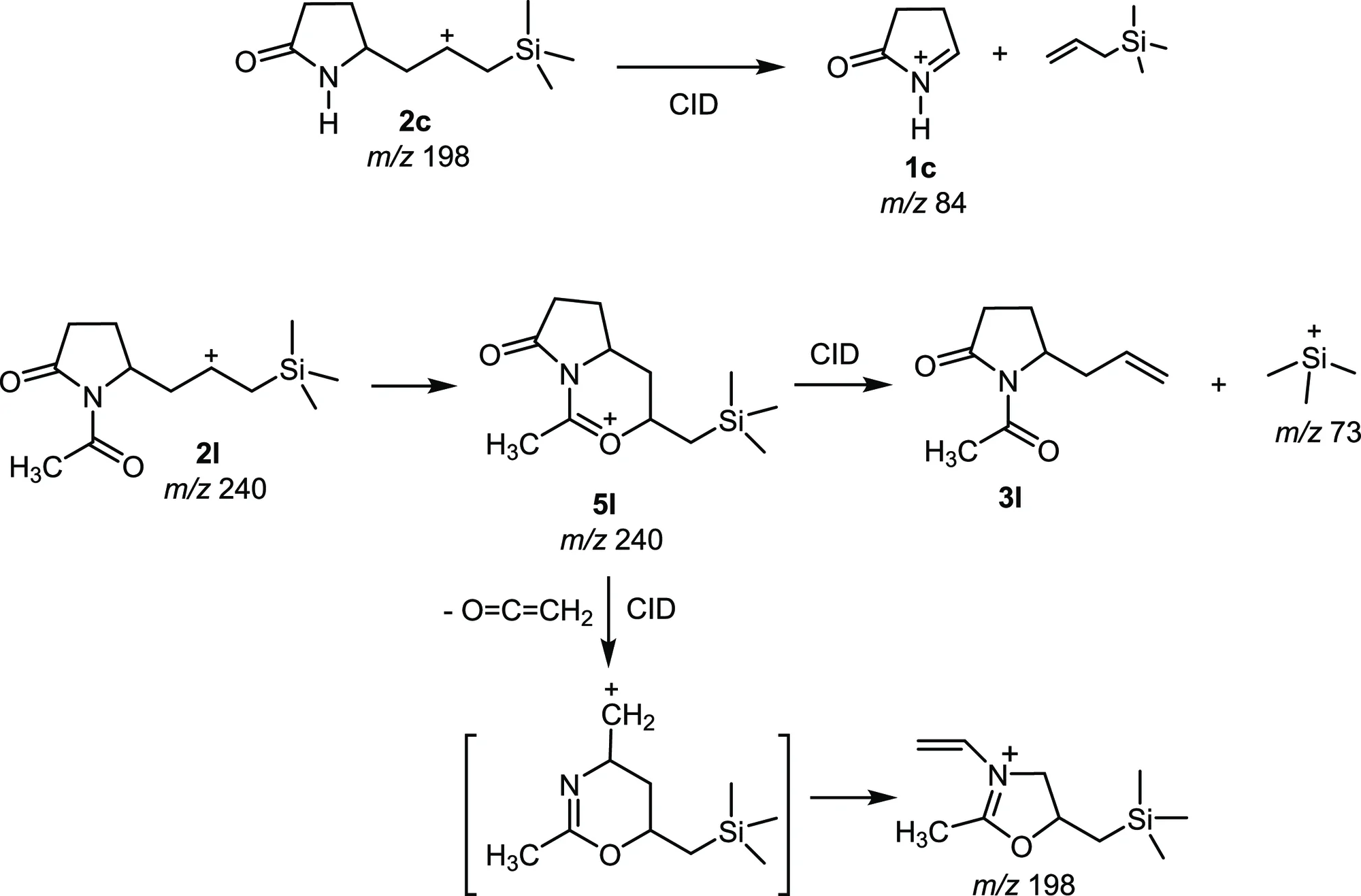
Proposed CID for the Mannich Intermediates **2c** and **2l** ^a^

a Note that cyclization of **2l** to **5l** prior to dissociation is assumed from the calculations, see Scheme 7 and the following discussions.

But the dissociation of the Mannich intermediate **2l** of *m*/*z* 240 formed from the *N,N*-diacyliminium ion **1l** shows no Grob fragmentation at all. Notably, **2l** fragments to two main product ions (Scheme 6), one of which being the Mannich product ion Si­(CH_3_)_3_^+^ of *m*/*z* 73. This fragmentation is equivalent to the whole course of a gas-phase Mannich reaction that forms both Si­(CH_3_)_3_^+^ and the neutral product **3l** (Scheme 2). The chemistry of **2l** provides therefore evidence for the covalent-bond nature of intermediates **2**.

To quickly access the thermodynamics of these reactions, we ran PM6 semiempirical calculations^16^ using the five-membered cyclic ions **1c**, **1m** and **1l** as examples (Figure 7). A major finding from these calculations was that all four intermediates **2l**–**o**, upon geometry optimization starting from their more stable *s-cis* conformation, collapse to the bicyclic intermediates **5l**–**o** (Scheme 7). This far greater stability of the bicyclic **5l**–**o** indicates therefore that the reactions of the four *N,N*-diacyliminium ions **1l**–**o** with ATMS proceed formally by polar [4^+^ + 2] cycloaddition (Scheme 7), as we observed for **1n** in reactions with vinyloxytrimethylsilane (Scheme 3).^9^

**Figure 7:**
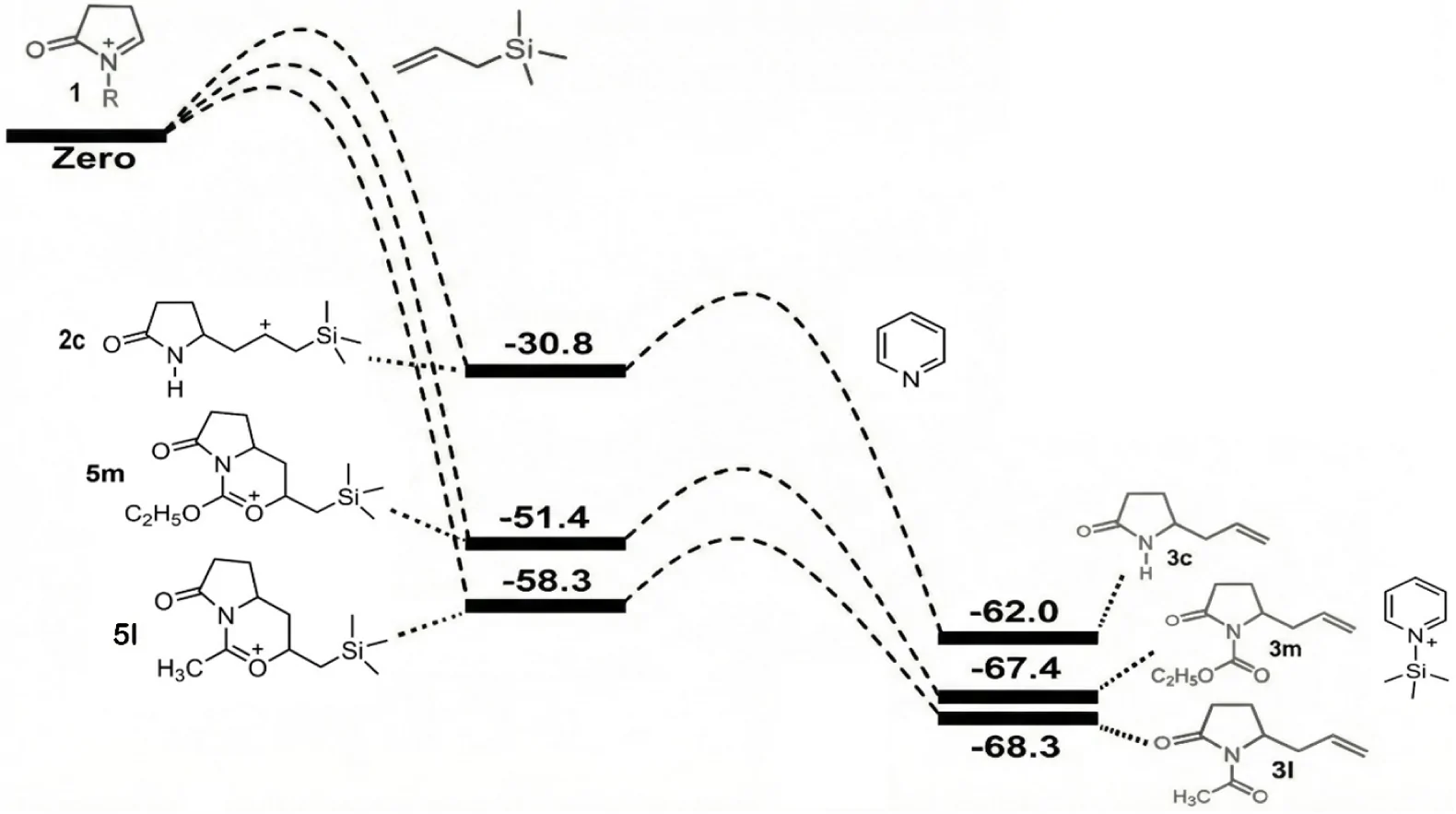
PM6 potential energy diagram for the Mannich reactions of the *N*-monoacyliminium ion **1c** and the *N,N*-diacyliminium ions **1m,l**. TS energies have not been calculated and are shown just as illustrations.

**Scheme 7:**
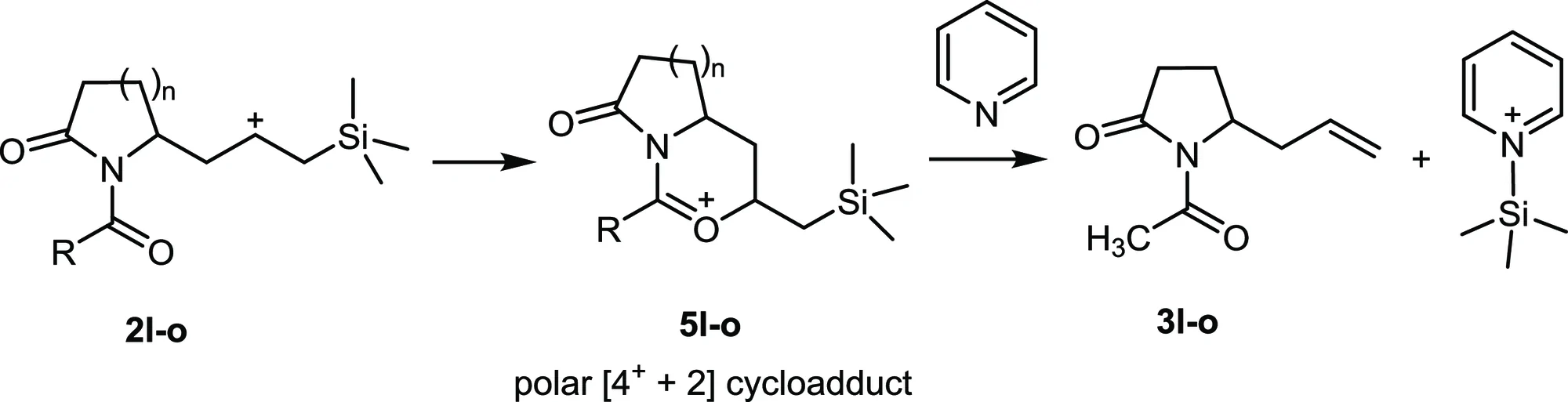
Spontaneous Cyclization of the Mannich Intermediates **2l**–**o** to the Polar [4^+^ + 2] Cycloadducts **5l**–**o** Indicated by the Calculations, and the Reactions of **5l**–**o** with a Model Nucleophile (Pyridine) to form the Final Mannich Products **3l**–**o**

Anyway, regardless of their actual structures **2l**–**o** or **5l**–**o**, these intermediates would be expected, upon nucleophile attack, to form the final Mannich products **3l**–**o** (Scheme 7). Note in Figure 7 that indeed **1l** and **1m** are predicted to form the polar [4^+^ + 2] intermediates **5l** (−58.3 kcal/mol) and **5m** (−51.4 kcal/mol) and final products **3l** (−68.3 kcal/mol) and **3m** (−67.4 kcal/mol) via far more exothermic reactions than that for **1c** that forms the single addition intermediate **2c** (−30.8 kcal/mol) and **3c** (−62.0 kcal/mol). For the calculations, to form the final Mannich products **3,** we used pyridine as a model nucleophile.

## Conclusion

Although they have remained elusive in solution, we have formed, isolated, and compared the intrinsic solvent- and counterion-free Mannich reactivity toward ATMS of four cyclic *N*,*N*-diacyliminium ions **1l**–**o** to that of their homologue *N*-monoacyliminium ions. The covalently bond and bicyclic nature of the intercepted Mannich intermediates **5l**–**o** has also been confirmed. Their bicyclic structures **5l**–**o**, predicted by calculations, seem also to indicate that the cyclic *N,N*-diacyliminium ions **1l**–**o** that we investigated, bearing endocyclic *N*-acyl groups and displaying favored *s-cis* conformation, react with ATMS via polar [4^+^ + 2] cycloadditions, as we had observed for **1n** in reactions with vinyloxytrimethylsilane. Having evaluated *N,N*-diacyliminium ions, we can now report a complete reactivity scale for cyclic nonacyl, *N*-monoacyl, and *N,N*-diacyliminium ions (Scheme 8), noting that indeed *N,N*-diacyliminium ions are far the most reactive, offering very attractive intermediates for Mannich-type transformations.

**Scheme 8:**
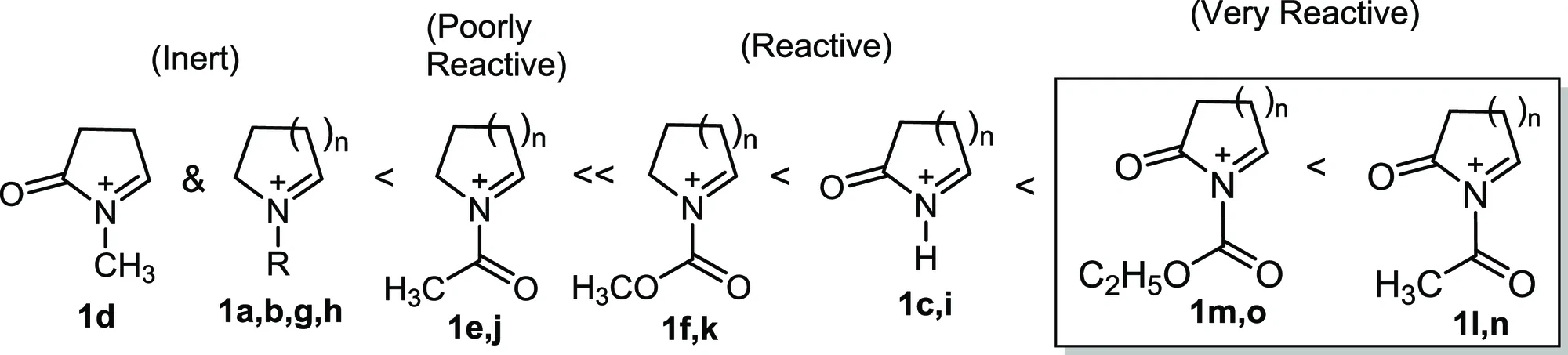
Reactivity Scale for Cyclic Non-Acyl, *N*-Monoacyl, and *N,N*-Diacyliminium Ions

*N,N*-Diacyliminium ions are the most reactive likely because the two *N*-acyl groups disfavor Grob fragmentation while increasing electrophilicity via resonance interaction with the nitrogen lone pair and electron-attracting inductive effects, thus favoring ATMS nucleophilic attack and addition at C2. The effect of the exocyclic *N*-acyl group is less effective due to facile rotation (an average C2–H Mulliken charge of +0.47 for **1e** as compared to +0.52 for **1c**), but in *N,N*-diacyliminium ions **1l**–**o** it sums up to the stronger effect of the endocyclic *N*-acyl group, which is locked in planar *s-trans* conformation.

Long ago, in a preliminary experiment, we were able to generate and react **1n** in solution with butyl vinyl ether, detecting by GC/MS what appears to be the final Mannich product. Unfortunately, more than two decades after data collection, we were unable to locate the original records. We nevertheless cite this undocumented but inspiring observation to motivate systematic explorations of the solution chemistry of the highly reactive, yet interceptable and highly synthetically attractive, *N,N*-diacyliminium ions.

## Supplementary Material



## Data Availability

The data underlying this study are available in the published article and its Supporting Information.

## Notes

MS^3^ for CID of **5n** (**2n**) and **5o** (**2o**) and proposed dissociation schemes for **5m**, **5n**, and **5o** (PDF)

## #

Institute for Creation Research, Dallas, TX 75229, USA
