# Supplementary material for: Intrinsic Reactivity of the Elusive Cyclic N,N‑Diacyliminium Ions in Mannich Reactions: The Complete Reactivity Order for Non, N‑Mono and N,N‑Diacyliminium Ions
Source: J Org Chem. 2026 Aug 4;91(32):10798–806. doi: 10.1021/acs.joc.6c00237 (PMC13488438; doi:10.1021/acs.joc.6c00237)
Supplement: Supplementary file 1 [file jo6c00237_si_001.pdf]

Intrinsic Reactivity of the Elusive Cyclic *N,N*-Diacyliminium Ions in Mannich Reactions: The Complete Reactivity Order for *Non*, *N*-Mono and *N,N*-Diacyliminium Ions.

Luiz Alberto B. de Moraes,[a] Adriano Reis,[b] Marcelo G. Montes D'Oca,[c] Cristina M. Schuch,[d] Ronaldo A. Pilli,[d] Rodinei Augusti[e] and Marcos N. Eberlin[b]*

SUPLEMENTARY MATERIAL

Table of Content

Page	Content
**S2**	**Figure S1**. MS^3 for CID of the Mannich intermediates **2n** of *m/z* 198 and **2o** of *m/z* 240. In fact, these ions likely collapse to their corresponding polar [4$^+$ + 2] cycloadducts **5n** and **5o**, see **Scheme 7**.
**S3**	**Scheme S1** Proposed dissociations for the Mannich intermediate **5m**.
**S3**	**Scheme S2** Proposed dissociations for the Mannich intermediate **5o**.
**S4**	**Scheme S3** Proposed dissociations for the Mannich intermediate **5o**.

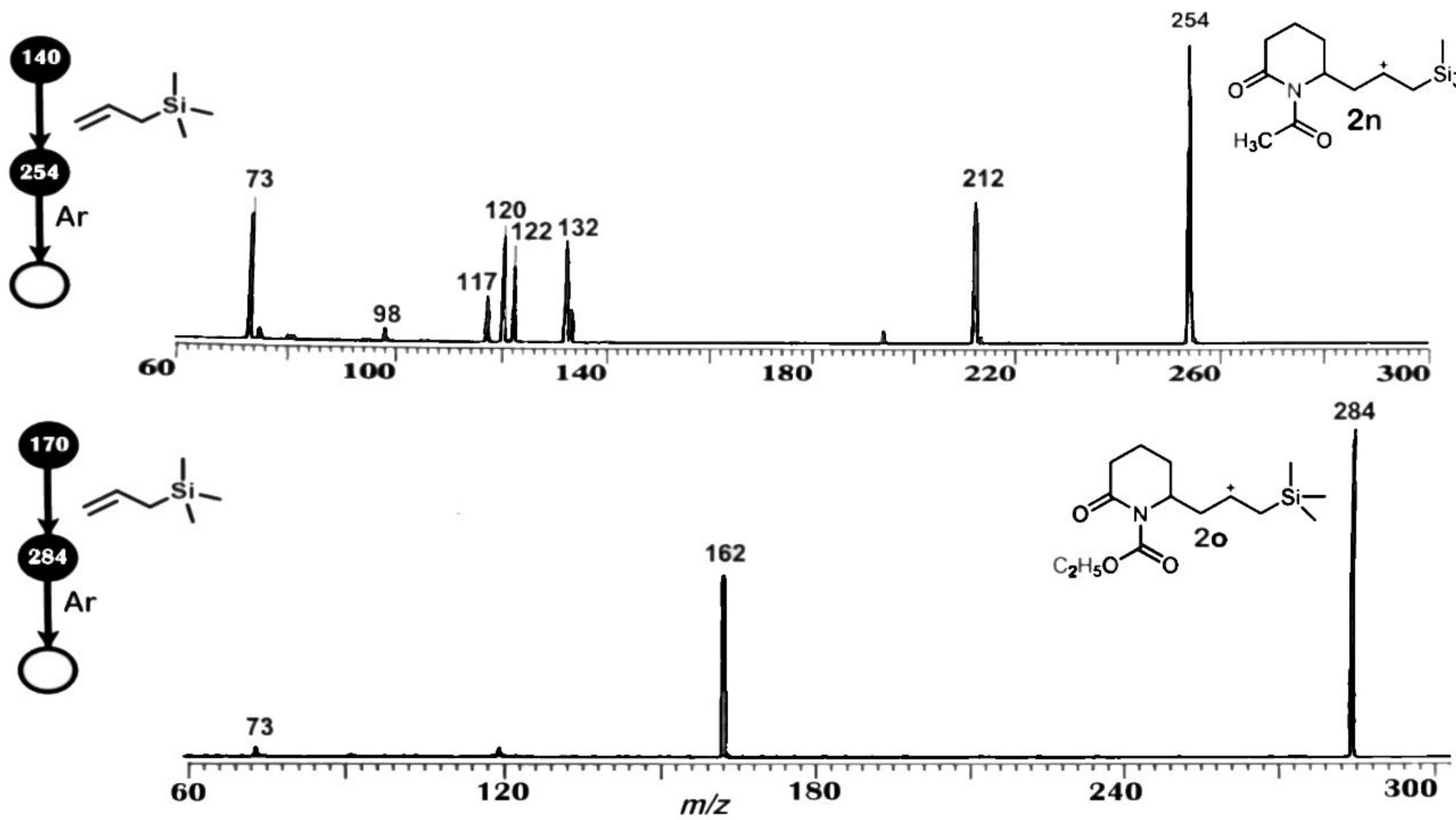

Figure S1. MS[3] for CID of the Mannich intermediates **2n** of *m/z* 198 and **2o** of *m/z* 240. In fact, these ions likely collapse to their corresponding polar [4$^+$ + 2] cycloadducts **5n** and **5o**, see **Scheme 7**.

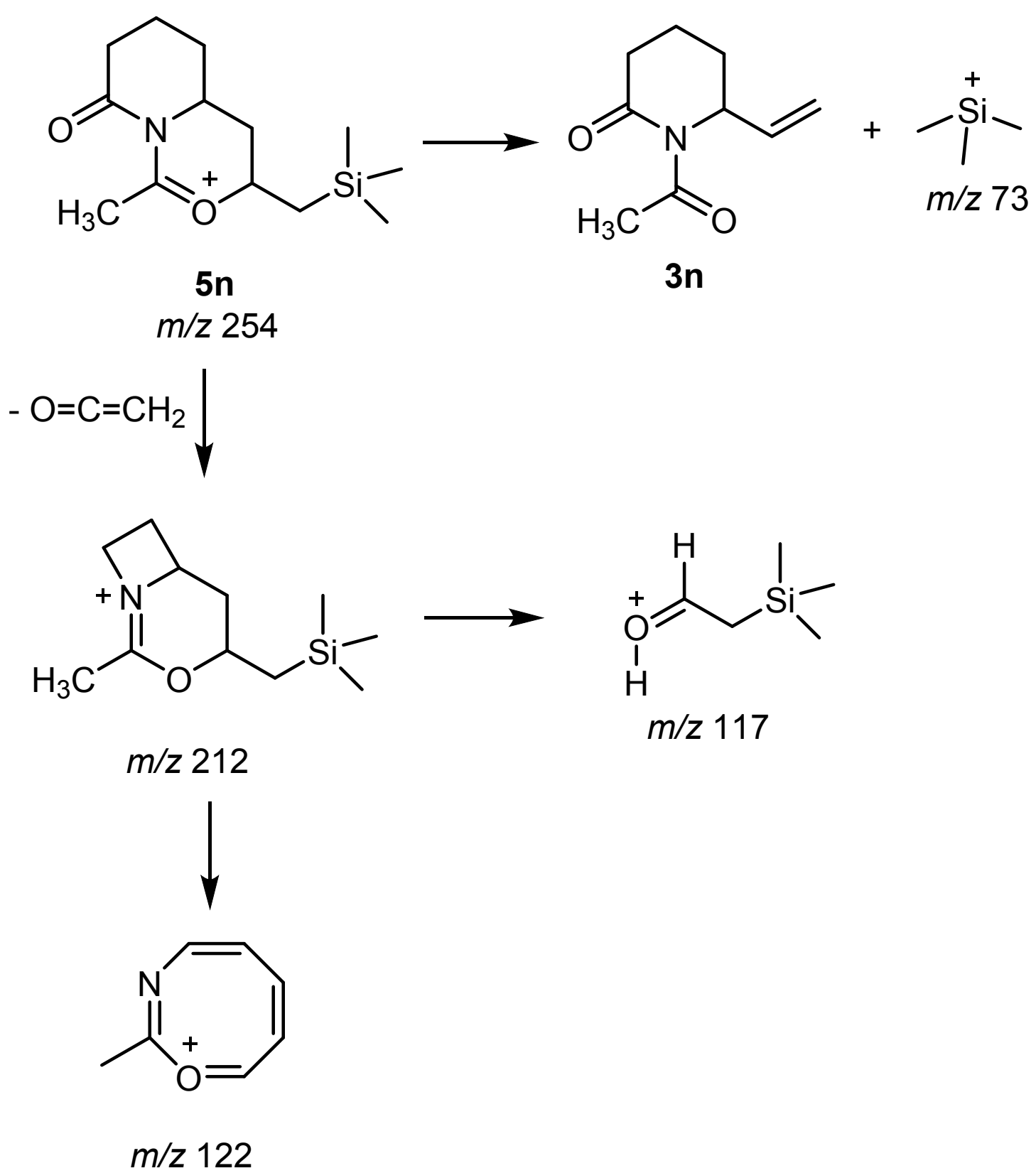

Scheme S1. Proposed dissociations for the Mannich intermediate **5m**.

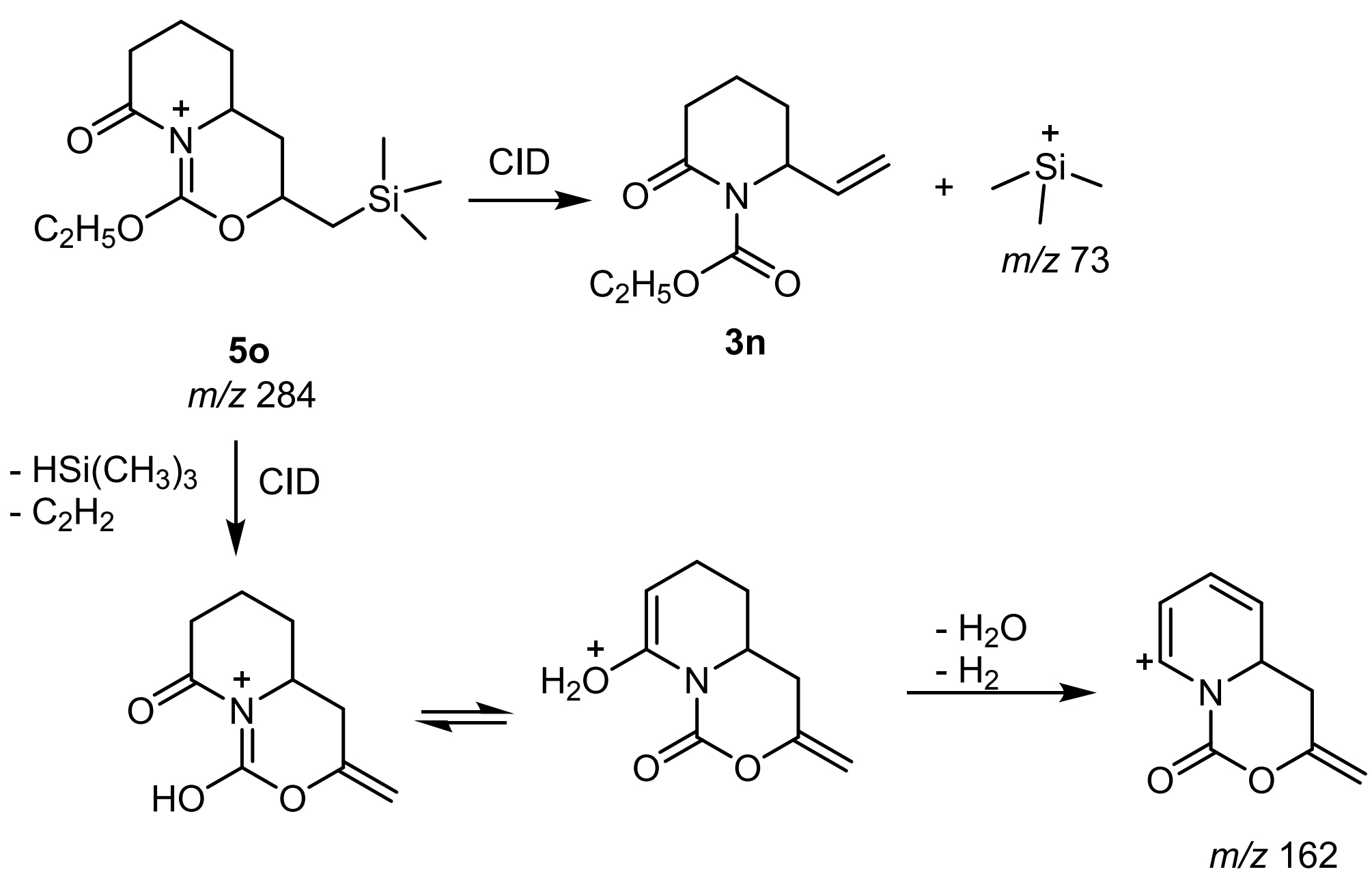

Scheme S2. Proposed dissociations for the Mannich intermediate **5o**.

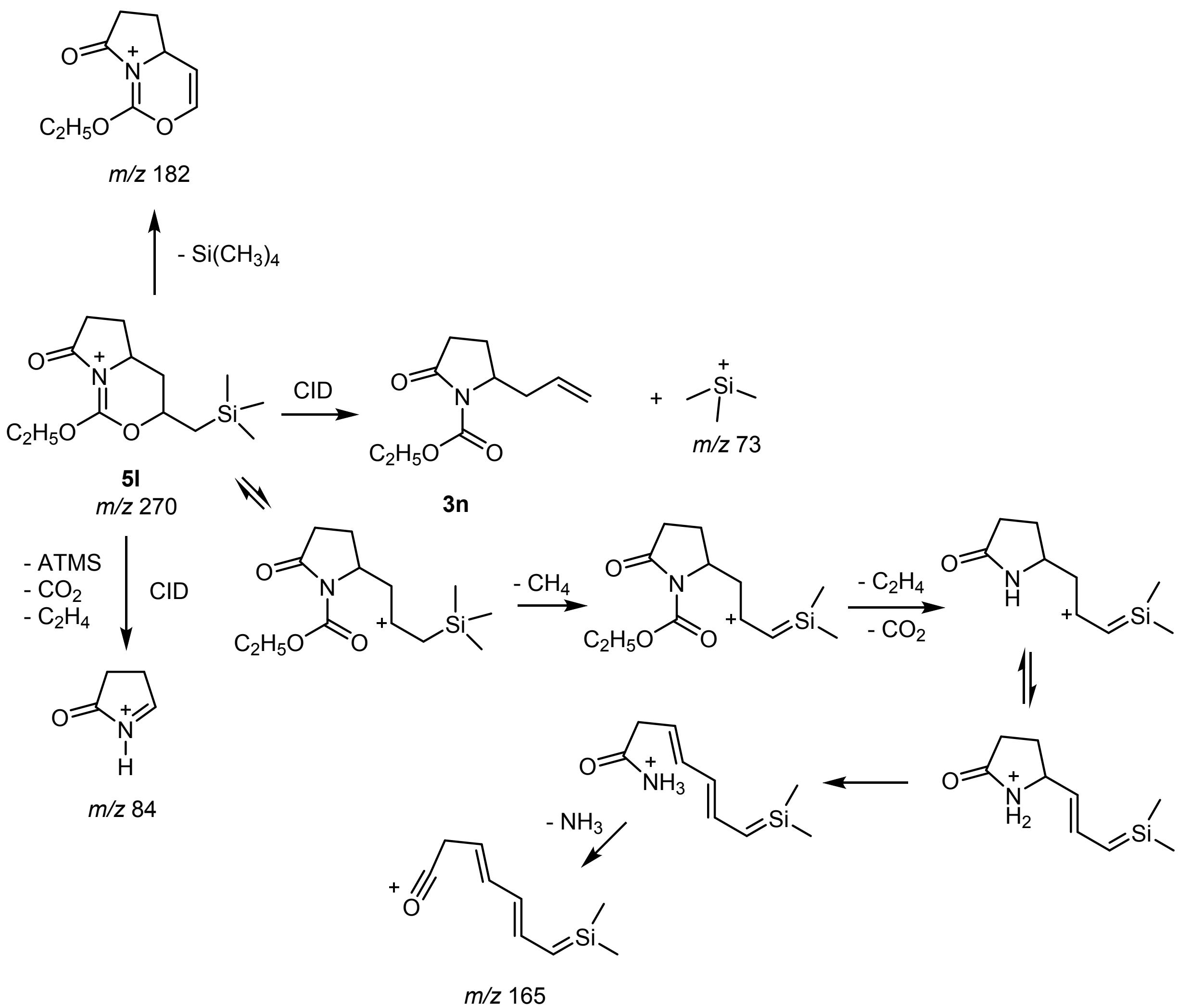

Scheme S3. Proposed dissociations for the Mannich intermediate **5o**.
